# Engagement as a binding agent: a critical analysis of the revised community of inquiry framework

**DOI:** 10.3389/fpsyg.2025.1666669

**Published:** 2026-02-09

**Authors:** Chengte Kuo

**Affiliations:** Department of Technology Application and Human Resource Development, National Taiwan Normal University, Taipei City, Taiwan

**Keywords:** blended learning, community of inquiry, engagement, learner presence, learning design, online learning

## Introduction

The digital transformation of education has fundamentally changed how learning communities are conceptualized in online and blended environments. The seminal Community of Inquiry (CoI) framework developed by Garrison et al. (1999) has provided a foundational three-dimensional model encompassing teaching, social, and cognitive presences. However, emerging research reveals a critical gap: how do these presences integrate to create effective learning ecosystems? The central problem lies not in identifying individual presences but in explicating the mechanisms through which they coalesce to produce meaningful learning outcomes in technology-mediated contexts.

This analysis addresses a fundamental theoretical question: How does engagement function as a binding agent that integrates the four presences—including the recently added learner presence—within the Revised Community of Inquiry (RCoI) framework in online and blended learning contexts? The addition of learner presence (Shea and Bidjerano, 2012; Armah et al., 2023) has introduced new complexities regarding how engagement operates within this expanded framework, revealing gaps in understanding how these four presences integrate in technology-enhanced learning settings.

## The revised community of inquiry framework: measurement challenges and theoretical gaps

Understanding the measurement of the CoI framework provides essential context for current challenges. Anderson et al. (2001) established methodological foundations for examining instructor facilitation in computer-mediated contexts. Building on this, Arbaugh et al. (2008) developed a validated comprehensive instrument operationalizing the original CoI framework across three core presences: teaching presence, social presence, and cognitive presence. This instrument became foundational for empirical research examining online learning.

Garrison and Akyol (2015) advanced the framework by incorporating metacognition as a critical construct, recognizing that learners‘ awareness and regulation of cognitive processes play essential roles across all presences. Richardson et al. (2017) conducted a meta-analysis demonstrating significant positive associations between social presence and students' satisfaction and learning in online environments, underscoring its importance as a predictor of both affective and cognitive outcomes.

The expansion to include learner presence shifts focus from instructor-centered and peer-centered interactions to individual learner agency and self-regulation. Shea et al. (2012, 2013) proposed learner presence as an additional conceptual element, emphasizing learners' self- and co-regulatory processes. Chang et al. (2025) conducted a systematic review of 26 quantitative studies (2010–2024) confirming that learner presence is reliably measurable and enhances the explanatory power of the CoI framework. However, their synthesis underscored a critical methodological challenge: current standard measurement instruments often inadequately capture the learner's active role, particularly concerning advanced dimensions such as co-regulation and peer facilitation. A refined conceptualization is required to address these deficiencies.

## Engagement as a binding agent: theoretical mechanisms

Despite advances in understanding individual presences, expanding to include learner presence necessitates a more nuanced conceptualization of how these four dimensions interact through engagement in technology-mediated environments. Current measurement approaches require expansion to capture the dynamic ways in which engagement integrates all four presences.

This analysis posits that engagement functions as a dynamic binding agent through two complementary mechanisms. First, engagement moderates the relationships between the four presences themselves, intensifying or attenuating direct connections among teaching, social, cognitive, and learner presences. Second, engagement moderates sequential mediational pathways through which these presences influence one another and ultimately produce learning outcomes. Unlike traditional additive models, where presences contribute independently, this binding agent framework suggests multiplicative effects wherein each presence's impact is contingent upon both the strength of engagement and the presence of other presences in the system.

This perspective finds support in empirical patterns. Chang et al. (2025) synthesized findings demonstrating that learner presence plays a dynamic relational role: it often mediates the effects of teaching and social presence on cognitive presence in technology-mediated learning. Empirical evidence indicates that learner presence exerts moderating effects, where the impact of traditional presences is contingent upon learners' regulatory capabilities. The binding agent model extends this understanding by formally conceptualizing engagement as a force that ensures system coherence by simultaneously moderating both direct presence-to-presence relationships and sequential mediational chains, thereby transforming disparate presence elements into a cohesive learning ecosystem.

## Student engagement: a multidimensional framework

Student engagement has emerged as a critical construct in educational research, conceptualized as a multidimensional rather than unitary phenomenon. Fredricks et al. (2004) established a comprehensive framework distinguishing three interconnected dimensions: behavioral engagement (participation in academic activities including attendance, effort, and persistence), emotional engagement (affective responses including sense of belonging, interest, and relationships with peers and teachers), and cognitive engagement (intellectual investment including metacognitive processes, strategic thinking, and willingness to embrace academic challenges).

Appleton et al. (2006) advanced this framework by developing the Student Engagement Instrument (SEI), providing empirical measurement tools to operationalize these dimensions. Skinner et al. (2008) demonstrated that engagement and disaffection constitute distinct motivational constructs rather than opposite ends of a continuum. This distinction suggests that interventions aimed at increasing engagement differ substantially from those addressing disaffection. These multidimensional engagement dimensions provide the foundation for understanding how engagement binds presences in online and blended learning environments.

## Engagement's integrative function: binding mechanisms across presences

Engagement's integrative function operates through distinct behavioral, emotional, and cognitive mechanisms that link specific presences. Rather than functioning in isolation, engagement creates observable moments where multiple presences converge simultaneously, producing multiplicative rather than additive effects.

### Learner presence binding

Learner presence binding occurs through emotional and behavioral mechanisms rooted in self-efficacy and self-regulation. When learners develop strong self-efficacy beliefs, cognitive engagement intensifies, making them simultaneously more responsive to teaching presence scaffolding and more willing to engage peers. A learner who perceives competence in online problem-solving is more likely to ask clarifying questions in discussion forums (behavioral engagement with social presence), apply instructor feedback iteratively (cognitive engagement with teaching presence), and persist through challenging tasks. Research by Alsayer et al. (2024) indicates that when effort regulation remains high, the cognitive demands of tasks forge stronger connections between individual thinking and peer collaboration, transforming behavioral engagement into affective investment.

### Teaching presence binding

Teaching presence binds through quality instructional guidance that creates opportunities for meaningful cognitive struggle within an emotionally safe space. Rather than transmitting content, teaching presence functions as a binding mechanism when instructors design scaffolding that simultaneously activates learner self-regulation and social collaboration. Providing partially worked-out examples that require students to complete the remaining steps, while encouraging peer consultation, creates conditions in which teaching guidance, cognitive challenge, and social interaction become inseparable. This integrated scaffolding generates behavioral evidence of engagement, reinforcing all connected presences.

### Cognitive presence binding

Cognitive presence binding operates through triggering metacognitive awareness that connects individual thinking processes with community knowledge construction. When learners engage in substantive discussions requiring explanation and argumentation, their cognitive processing becomes observable through their discussion contributions, simultaneously serving both personal meaning-making and community learning. Cho et al. (2017) demonstrated that when effort regulation remains high, complex tasks create stronger connections between individual processing and peer collaboration, strengthening the binding function.

High-binding moments exemplify meaningful synthesis across presences: a student response that simultaneously applies an instructor strategy to their own problem, seeks peer input on implementation, demonstrates metacognitive awareness, and shows emotional investment in the collaborative solution, evidencing integration rather than isolated mastery of component skills.

### Social presence binding

Social presence binds functions through emotional resonance and empathetic connection, sustaining engagement during interpersonal challenges. When community members respond to contributions with genuine engagement rather than perfunctory acknowledgment, learners develop affective investment that increases willingness to take collaborative risks—asking peers for help, sharing uncertainties, or proposing alternative perspectives. This emotional bond creates behavioral commitment to community participation, which feeds back into cognitive and teaching presence through increased responsiveness to guidance and deeper collaborative processing, establishing a reinforcing cycle.

## Boundary conditions: culture and institution

The effectiveness of the binding function is significantly shaped by cultural values and institutional conditions, requiring contextual calibration. Engagement's binding mechanisms do not operate uniformly across all contexts; they manifest differently depending on how cultural and structural factors modulate presence dynamics.

### Cultural alignment

Cultural alignment represents a critical boundary condition. Cho et al. (2017) demonstrated that when learners employ self-regulation strategies aligned with their cultural backgrounds—such as explicit strategy awareness in individualistic contexts vs. implicit alignment with group norms in collectivistic contexts—engagement more effectively facilitates coordination among presences. This suggests that binding mechanisms must be culturally responsive rather than culturally neutral. Discussion forum scaffolding that encourages rapid individual idea generation may disrupt binding in contexts where consensus-building and thoughtful reflection constitute culturally congruent engagement patterns.

### Institutional context and infrastructure

The institutional context similarly shapes binding capacity. Zhang and Zhu (2023) found that institutional presence—encompassing technological infrastructure, technical support systems, and administrative policies—functions as a conditional moderator of the relationship between learner presence and engagement outcomes, with effects observed across 762 participants from multiple institutions. Well-resourced institutions with responsive technical support enabled engagement that more effectively bound learner capabilities to pedagogical intentions than under-resourced contexts, where technical barriers fragmented learner attention. These findings position institutional presence not as a fifth presence element but as a structural enabler of binding effectiveness.

## Concrete design and evaluation implications

The binding agent framework generates three specific implications for learning design and assessment that prioritize integration over component optimization.

### Early scaffolding of effort regulation

Since behavioral engagement moderates inter-presence relationships, it should be established before content demands peak. Courses should explicitly teach and scaffold self-regulation strategies in opening modules—such as requiring students to identify personal effort-regulation triggers through guided reflection, practice applying chosen strategies in low-stakes collaborative tasks, and establish peer accountability partnerships for maintaining effort during challenging periods. This proactive scaffolding creates early binding by ensuring engagement mechanisms are activated before cognitive and social demands peak.

### Identification of “binding moments” via trace data

Effective learning creates observable behaviors where multiple presences converge simultaneously. Evaluation should shift from measuring isolated presences to capturing binding moments. Learning management system trace data—timestamps, response sequences, and revision patterns—can identify single behaviors demonstrating simultaneous integration, such as a post that applies an instructor strategy, seeks peer input, and demonstrates metacognitive awareness. These data enable instructors to recognize and reinforce binding effectiveness in real-time.

### Assessment of integration quality

Assessment criteria should evaluate synthesis across presences rather than grading components in isolation. Integration-quality rubrics assess the degree to which a student's contribution integrates peer perspectives with personal regulatory processes. A group project rubric could evaluate not only final product quality and individual contributions but also the degree to which contributions show adaptive responses to feedback, integration of peer perspectives, and evidence that individual effort supported collective coherence—dimensions that reveal binding effectiveness rather than mere component execution.

## Discussion

### Theoretical contributions

The conceptualization of engagement as a binding agent offers a starting point for reconsidering how the RCoI framework achieves educational effectiveness in online and blended learning environments. Unlike traditional additive models that treat presence as an independent variable, this perspective suggests that engagement may create emergent properties by dynamically integrating teaching, social, cognitive, and learner presences into a coherent learning ecosystem.

This conceptualization addresses a gap in existing CoI literature: while previous research established the importance of individual presences, less attention has been given to explaining how these presences combine to produce learning effects. The binding agent perspective proposes that engagement might function as an integrative force, weaving disparate presence elements into unified learning experiences, though this proposition requires substantial empirical validation.

The framework suggests that engagement may operate through multiplicative rather than additive effects—potentially amplifying interconnections between presences and creating feedback loops that generate emergent learning properties. Specifically, engagement might simultaneously moderate both direct presence-to-presence relationships and sequential mediational pathways. However, whether engagement indeed functions as this critical determinant of learning environment coherence remains an empirical question warranting systematic investigation.

### Future research directions

The binding agent framework opens specific avenues for testing and refining these theoretical propositions. Empirical validation using structural equation modeling with interaction effects and dynamic network analysis could provide evidence for engagement's potential moderating functions in both direct interpersonal relationships and sequential meditational chains.

Longitudinal research would be valuable for investigating how binding effectiveness evolves throughout technology-mediated learning experiences, identifying critical periods when integration strengthens or weakens. Cross-cultural investigations comparing engagement mechanisms across individualistic and collectivistic cultures are particularly important in global digital education contexts. Chang et al. (2025) found that manifestations of learner presence vary significantly across cultural contexts, suggesting that engagement's integrative role may also operate differently across cultures. Tripon (2025) emphasizes that integrating mentorship and community support is fundamental to sustaining educational quality in digital environments.

Controlled experiments comparing traditional component-optimization interventions with binding-enhancement interventions provide practical evidence for evaluating the utility of this framework. Measurement development represents a critical priority; current assessment instruments measure engagement and individual presence separately. Testing the binding agent framework requires developing tools that capture engagement's proposed integrative properties, the quality of presence connections, and the affective-relational dimensions that sustain coherent learning communities.

### Limitations

This analysis acknowledges several important limitations. First, the theoretical development relies on a conceptual synthesis of the existing literature rather than on an original empirical investigation specifically designed to test binding mechanisms. The proposed framework remains speculative until directly validated through systematic empirical research. Second, the complexity of hypothesized moderated relationships presents significant analytical challenges, requiring sophisticated statistical techniques and potentially novel methodological approaches. Third, implementation challenges may arise from the framework's emphasis on system-level thinking, which could conflict with established component-focused educational practices.

Despite these limitations, the binding agent framework offers a valuable conceptual foundation for future investigations into engagement's role in creating coherent technology-enhanced learning experiences through the dynamic integration of presence.

## Conclusion

This conceptual analysis has proposed engagement as a binding agent within the Revised Community of Inquiry framework, suggesting a mechanism through which teaching, social, cognitive, and learner presences might integrate to create coherent learning experiences in online and blended environments. The framework offers three primary contributions.

First, it provides a conceptual foundation for moving beyond additive models toward understanding potential multiplicative and emergent effects on technology-mediated learning. Second, it identifies specific mechanisms through which different engagement dimensions—behavioral, emotional, and cognitive—might link particular presences, with particular attention to self-regulation, metacognition, and emotional resonance as key processes. Third, this analysis highlights critical boundary conditions, particularly cultural alignment and institutional infrastructure, emphasizing that effective implementation requires careful contextual adaptation rather than universal, standardized application.

However, substantial work remains. The proposed framework is fundamentally speculative, requiring rigorous empirical validation before establishing utility. Future research must develop measurement instruments capturing engagement's hypothesized integrative properties, conduct longitudinal investigations across diverse cultural and institutional contexts, and employ sophisticated analytical techniques to test the complex moderated relationships proposed herein.

Ultimately, whether engagement truly functions as a binding agent in the Revised Community of Inquiry framework remains an open question. This analysis offers a starting point for systematic investigation of this possibility, with the hope that sustained research efforts will clarify engagement's role in creating effective technology-enhanced learning environments.

## References

[B1] Alsayer A. A. Templin J. Niileksela C. Frey B. B. (2024). Examining the structure of the revised community of inquiry framework: a multi-level approach. Educ. inf. technol. 30, 6785–6807. doi: 10.1007/s10639-024-13090-3

[B2] Anderson T. Rourke L. Garrison D. R. Archer W. (2001). Assessing teaching presence in a computer conferencing context. J. Asynchron. Learn. Netw. 5, 1–17. doi: 10.24059/olj.v5i2.1875

[B3] Appleton J. J. Christenson S. L. Kim D. Reschly A. L. (2006). Measuring cognitive and psychological engagement: validation of the student engagement instrument. J. Sch. Psychol. 44, 427–445. doi: 10.1016/j.jsp.2006.04.002

[B4] Arbaugh J. B. Cleveland-Innes M. Diaz S. R. Garrison D. R. Ice P. Richardson J. C. . (2008). Developing a community of inquiry instrument: testing a measure of the Community of Inquiry framework. Internet High. Educ. 11, 133–136. doi: 10.1016/j.iheduc.2008.06.003

[B5] Armah J. K. Bervell B. Bonsu N. O. (2023). Modelling the role of learner presence within the community of inquiry framework to determine online course satisfaction in distance education. Heliyon 9:e15803. doi: 10.1016/j.heliyon.2023.e15803 37180887 PMC10172789

[B6] Chang C.-C. Yen W.-H. Liu S.-C. Kuo C.-T. (2025). Learning presence in the revised community of inquiry framework: a systematic review of empirical research (2010–2024). Internet High. Educ. 68:101058. doi: 10.1016/j.iheduc.2025.101058

[B7] Cho M. H. Kim Y. Choi D. (2017). Investigation of the community of inquiry framework regarding self-regulation, metacognition, and motivation. Comput. Educ. 126, 53–64. doi: 10.1016/j.compedu.2018.06.032

[B8] Fredricks J. A. Blumenfeld P. C. Paris A. H. (2004). School engagement: potential of the concept, state of the evidence. Rev. Educ. Res. 74, 59–109. doi: 10.3102/00346543074001059

[B9] Garrison D. R. Akyol Z. (2015). Toward the development of a metacognition construct for the community of inquiry framework. Internet High. Educ. 24, 66–71. doi: 10.1016/j.iheduc.2014.10.001

[B10] Garrison D. R. Anderson T. Archer W. (1999). Critical inquiry in a text-based environment: computer conferencing in higher education. Internet High. Educ. 2, 87–105. doi: 10.1016/S1096-7516(00)00016-6

[B11] Richardson J. C. Maeda Y. Lv J. Caskurlu S. (2017). Social presence in relation to students' satisfaction and learning in the online environment: a meta-analysis. Comput. Human Behav. 71, 402–417. doi: 10.1016/j.chb.2017.02.001

[B12] Shea P. Bidjerano T. (2012). Learning presence as a moderator in the community of inquiry model. Comput. Educ. 59, 316–326. doi: 10.1016/j.compedu.2012.01.011

[B13] Shea P. Hayes S. Smith S. U. Vickers J. Bidjerano T. Pickett A. . (2012). Learning presence: additional research on a new conceptual element within the community of inquiry framework. Internet High. Educ. 15, 89–95. doi: 10.1016/j.iheduc.2011.08.002

[B14] Shea P. Hayes S. Uzuner-Smith S. Vickers J. Bidjerano T. Gozza-Cohen M. . (2013). Online learner self-regulation: learning presence, viewed through quantitative content- and social network analysis. Int. Rev. Res. Open Distrib. Learn. 14, 427–461. doi: 10.19173/irrodl.v14i3.1466

[B15] Skinner E. Furman M. J. Marchand G. Kindermann T. (2008). Engagement and disaffection in the classroom: part of a larger motivational dynamic? J. Educ. Psychol. 100, 765–781. doi: 10.1037/a0012840

[B16] Tripon C. (2025). Towards quality education for all: integrating EdTech, mentorship, and community in support of SDG 4. Educ. Sci. 15:1184. doi: 10.3390/educsci15091184

[B17] Zhang W. Zhu C. (2023). Institutional presence: toward a further developed Community of Inquiry model integrating institutional functions in online and blended learning environments. Front. Psychol. 14:1132204. doi: 10.3389/fpsyg.2023.1132204 36993888 PMC10040635

